# Supplementary material for: Molecular diversity of Giardia duodenalis in children under 5 years from the Manhiça district, Southern Mozambique enrolled in a matched case-control study on the aetiology of diarrhoea
Source: PLoS Negl Trop Dis. 2021 Jan 19;15(1):e0008987. doi: 10.1371/journal.pntd.0008987 (PMC7846004; doi:10.1371/journal.pntd.0008987)
Supplement: S1 Table — (DOCX) [file pntd.0008987.s001.docx]

**S1 Table. List and sequence of primers used for molecular identification of *G. duodenalis* assemblages and multilocus sequence genotyping**

| **Designation** | **Primer** | **Sequence (5’–3’)** | **Amplicon size (bp)** | **Reference** |
| --- | --- | --- | --- | --- |
| Multiplex I  (*E1-HP*) | 4E1-HP-A_F | AAAGAGATAGTTCGCGATGTC | 165 | [1] |
|  | 4E1-HP-A_R | ATTAACAAACAGGGAGACGTATG |  | [1] |
|  | 4E1-HP-B_F | GAAGTCATCTCTGGGGCAAG | 272 | [1] |
|  | 4E1-HP-B_R | GAAGTCTAGATAAACGTGTCGG |  | [1] |
| Multiplex II  (*C1-P21*) | 5C1-P21-A_F | ATGCTAGCCGTAGTTAATAAGG | 303 | [1] |
|  | 5C1-P21-A_R | ACCGGCCTTATCTACCAGC |  | [1] |
|  | 5C1-P21-B_F | TTAATAGAAATGCTTTCGACACG | 249 | [1] |
|  | 5C1-P21-B_R | TTGCTACAGCAGAAAGGTGC |  | [1] |
| *gdh* | GDHe_F | TCAACGTYAAYCGYGGYTTCCGT | 432 | [2] |
|  | GDHi_R | GTTRTCCTTGCACATCTCC |  | [2] |
|  | GDHi_F | CAGTACAACTCYGCTCTCGG |  | [2] |
| *β-giardin* | G7_F | AAGCCCGACGACCTCACCCGCAGTGC | 511 | [3] |
|  | G759_R | GAGGCCGCCCTGGATCTTCGAGACGAC |  | [3] |
|  | G99_F | GAACGAACGAGATCGAGGTCCG |  | [3] |
|  | G609_R | CTCGACGAGCTTCGTGTT |  | [3] |
| *tpi* | AL3543_F | AAATIATGCCTGCTCGTCG | 530 | [4] |
|  | AL3546_R | CAAACCTTITCCGCAAACC |  | [4] |
|  | AL3544_F | CCCTTCATCGGIGGTAACTT |  | [4] |
|  | AL3545_R | GTGGCCACCACICCCGTGCC |  | [4] |

**References**

1. Vanni I, Cacciò SM, van Lith L, Lebbad M, Svärd SG, Pozio E, et al. Detection of Giardia duodenalis assemblages A and B in human feces by simple, assemblage-specific PCR assays. PLoS Negl Trop Dis. 2012;6(8):1–9.

2. Read CM, Monis PT, Thompson RCA. Discrimination of all genotypes of Giardia duodenalis at the glutamate dehydrogenase locus using PCR-RFLP. 2004;4:125–30.

3. Lalle M, Pozio E, Capelli G, Bruschi F, Crotti D, Cacciò SM. Genetic heterogeneity at the β-giardin locus among human and animal isolates of Giardia duodenalis and identification of potentially zoonotic subgenotypes. Int J Parasitol. 2005;35(2):207–13.

4. Sulaiman IM, Fayer R, Bern C, Gilman RH, Trout JM, Schantz PM, et al. Triosephosphate Isomerase gene characterization and potential zoonotic transmission of Giardia duodenalis. Emerg Infect Dis. 2003;9(11):1444–52.
