# Supplementary figures and images for: Molecular diversity of Giardia duodenalis in children under 5 years from the Manhiça district, Southern Mozambique enrolled in a matched case-control study on the aetiology of diarrhoea
Source: PLoS Negl Trop Dis. 2021 Jan 19;15(1):e0008987. doi: 10.1371/journal.pntd.0008987 (PMC7846004; doi:10.1371/journal.pntd.0008987)

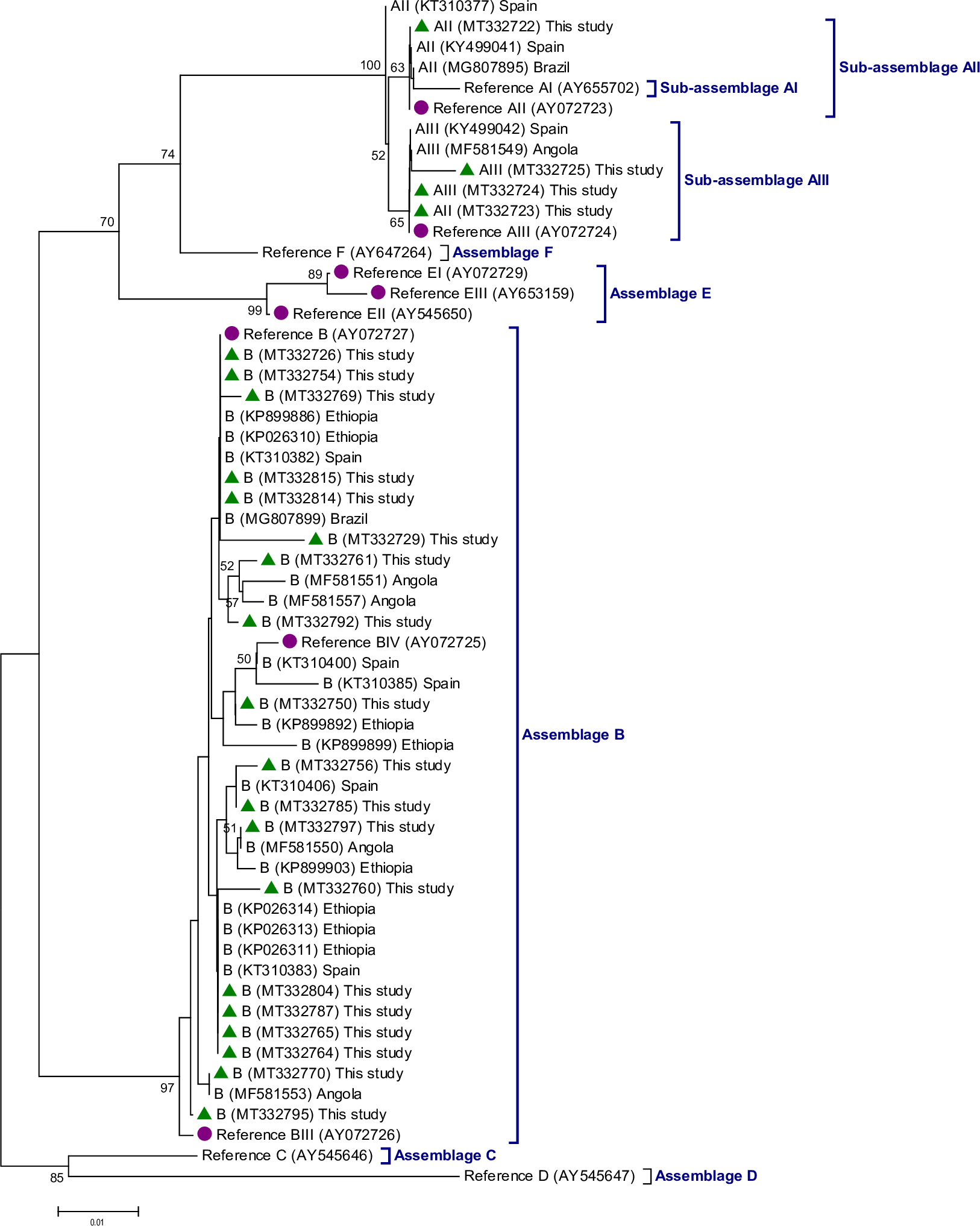

Supplement: S1 Fig — The analysis was inferred using the Neighbor-Joining method of the nucleotide sequence covering a 517-bp region (positions 96–612 of GenBank: AY072727) of the gene. Bootstrap values lower than 50% are not shown. Cyan filled circles represent reference sequences downloaded from the GenBank database; filled dark green triangles represent sequences generated in the present study. No outgroup sequence was used as bg is a Giardia-specific gene. (TIF) [file pntd.0008987.s007.tif]

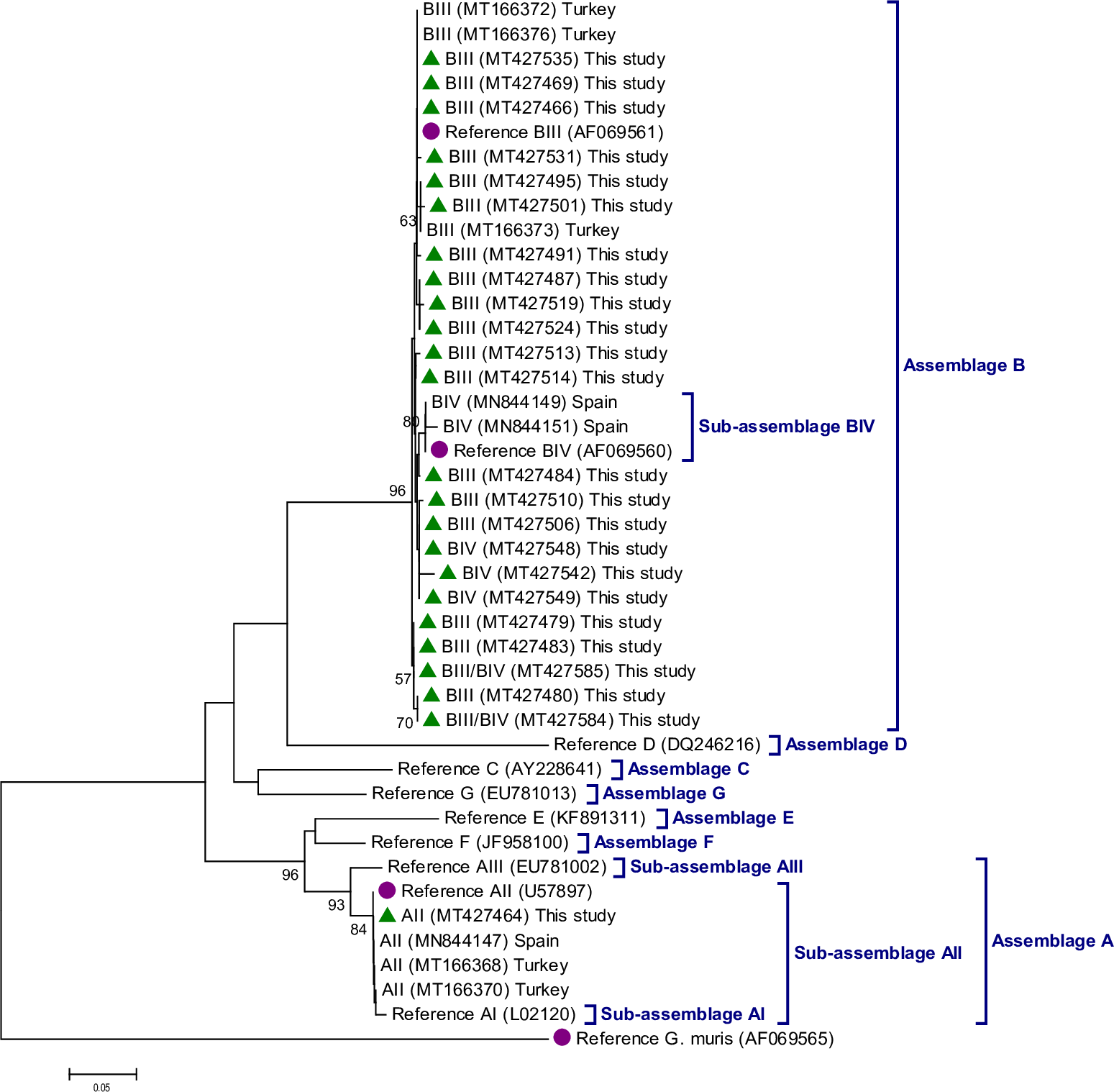

Supplement: S2 Fig — The analysis was inferred using the Neighbor-Joining method of the nucleotide sequence covering a 479-bp region (positions 1–479 of GenBank: AY069560) of the gene. Bootstrap values lower than 50% are not shown. Cyan filled circles represent reference sequences downloaded from the GenBank database; filled dark green triangles represent sequences generated in the present study. Giardia muris was used as the outgroup. (TIF) [file pntd.0008987.s008.tif]
