# Supplementary material for: Molecular diversity of Giardia duodenalis in children under 5 years from the Manhiça district, Southern Mozambique enrolled in a matched case-control study on the aetiology of diarrhoea
Source: PLoS Negl Trop Dis. 2021 Jan 19;15(1):e0008987. doi: 10.1371/journal.pntd.0008987 (PMC7846004; doi:10.1371/journal.pntd.0008987)
Supplement: Attachment — Submitted filename: Responses_to_reviewer_PNTD_D_20_01460R1.docx [file pntd.0008987.s009.docx]

**Molecular diversity of *Giardia duodenalis* in children under 5 years from the Manhiça district, Southern Mozambique enrolled in a matched case-control study on the aetiology of diarrhoea**

Subject : Manuscript PNTD-D-20-01460 by Messa et al.

Dear Dr. Mejia and Dr. Singer,

Thank you all for your favourable preliminary appraisal on Ms. PNTD-D-20-01460, and for making available to us the Reviewers' comments, which we have considered carefully. Please see below a thorough point-by-point response to each issue raised by Reviewers #1 and #2. Changes introduced in the main body of the text have been highlighted in red for better identification.

**Reviewer´s #1 comments**

In this study, it is proposed to establish the correlation between the symptoms (diarrhoea) and the genotype of the *Giardia* causing the infection. The results indicate that the relationship between the two is neither obvious nor significant, however they find a great genetic diversity among isolates of genotype B, based on previous works (Infection, Genetics and Evolution Volume 47, January 2017, Pages 155-160).

*Response: We thank Reviewer #1 for his/her preliminary positive appraisal of the merits of our study.*

1. They mention that mainly in children this one finds genotype B, while in adults genotype A is found more frequently, however the studies carried out in this research only include samples from children.

*Response: We thank the reviewer for the comments. We would like to clarify that the present study only focused on children younger than five years of age (see current lines 164-174 of the Methods section). No adult population was investigated. Please also note that both assemblages A and B were detected in the children population investigated (see current Table 3).*

2. They must specify from where they take the characteristics to classify the samples in the BIII / BIV subgenotypes.

*Response: We would like to clarify that the confirmation of G. duodenalis assemblages and assignment of sequences to specific sub-assemblages was conducted by multiple sequence alignment analyses with appropriate reference sequences using MEGA 6 software. This information is clearly stated in current lines 267-277 of the Methods section. Please also note that S3-S5 Tables show the details of each representative sequence identified in the present study at the three loci investigates, including the reference sequence used, the compared stretch, the presence of SNPs and if they were involved in amino acid change at the protein level.*

3. According to the methodology they carry out, is it possible that the ambiguities in the classification are due to the presence of other genotypes (C-H) that are not searched with this procedure?

*Response: We thank the reviewer for this point, however we do not believe that this is certainly the case. Please note that the initial assignment of assemblages in this survey was based on the results obtained by conventional multiplex PCR (see current lines 196-208 and our answer to comment #3 by Reviewer #2). This method was specifically developed to detect (and differentiate among) assemblages A and B, the two G. duodenalis genetic variants predominant in humans. In practical terms this means that detection of assemblages C-H were highly unlikely. Indeed, please note that we have acknowledged this fact as a potential limitation of our study (see lines 566-570 of the Discussion section).*

4. The presence of double peaks in the electropherogram can be removed with further purification of the genetic material? This would reduce the ambiguities in the classification

*Response: We agree with Reviewer #1. If the presence of double peaks during chromatogram inspection is due to true mixed infections involving different assemblages or sub-assemblages of the parasite, this issue can be resolved following two different approaches:*

i) *Reanalysing all G. duodenalis-positive samples using a next generation sequencing approach targeting the 18S locus of the parasite. This method is very sensitive and able to detect circulating genotypes that are underrepresented in the surveyed human population, allowing the discrimination of mixed infections that would be undetectable by Sanger sequencing.*
ii) *Sequence analysis of multiple cloned PCR products using E. coli plasmid vectors.*

*Unfortunately both methodologies are currently unavailable in our laboratory. We have acknowledged this limitation of the study in lines 570-575 of the Discussion section.*

5. The research aims to study the relationship between digestive pathology (diarrhea) in children and the presence of the duodenal *Giardia* genotype in their stools. According to the information presented, they do not find statistically significant differences between genotype and diarrhoea in children. The results show that they only found type B. It is striking that group A only appears in adults. According to the title of the work, it was planned to work only children because they included adults, would it be convenient to explain what the reason for doing it was. The exposed bibliography is updated. However, it does not speak of genetic diversity where it finds the BIII / BIV groups with results of double peaks that are interpreted as ambiguities and that allows them to think that they are different genotypes. From what I consider, it must present a difference in the electrophoretic shift electropherogram that supports this work as it is not well documented solidly. We must remember the genomic plasticity of *Giardia* makes it an interesting protozoan.

*Response: We would like to clarify that the present study only focused on children younger than five years of age (see current lines 170-177 of the Methods section). No adult population was investigated.*

*Regarding ambiguous BIII/BIV sequences please note that this finding may be due to true mixed infections or the occurrence of genetic recombination events. Both possibilities are fully discussed in our detailed explanation to comment #12*.

6. They do not mention the hypothesis of the work that should be included if they seek to relate the presence of diarrhoea and the *Giardia* genotype if they want to postulate the genotypes with the presence of diarrhoea in children.

*Response: We would like to clarify that the justification of the present study is clearly stated in lines 100-103 and lines 135-139 of the Introduction section. We feel this information is enough to formulate the hypothesis that the genotype of G. duodenalis may (or may not) determine the virulence and pathogenicity of the infection.*

7. The size of the sample is sufficient. However, they do not explain how it is calculated, one of the parameters that must be considered is the prevalence of Giardiasis in their country.

*Response: The authors would like to clarify that in the present work we retrospectively characterized at molecular level the positive samples for Giardia among children enrolled in the Global Enteric Multicenter study (GEMS) in Mozambique. All the information regarding the design of the GEMS project including sampling size, inclusion/exclusion criteria, geographical location, diagnostic methods used, sociodemographic and clinical data gathered, etc, are thoroughly described in references #36 and #42. Due to space constricts, the interested reader is invited to check these primary sources in current line 155-184 of the manuscript.*

8. They include statistical treatment where it is shown that no relationship between the parameters studied.

*Response: We thank Reviewer #1 for his/her positive assessment on this matter.*

9. There are no ethical problems at work

*Response: We thank Reviewer #1 for his/her positive assessment on this matter.*

10. The use of an enzymatic method to demonstrate the presence of the parasite is not conclusive because it is showing two different parametrons in the enzymatic method the presence of antibodies that is not always conclusive because it depends on the antigenic of the parasite's proteins. You must put the coproparasitoscopic in where the presence of the parasite is demonstrated. This is probably due to the different positivity when the gdhtpibg gene initiators were used, the results are shown in Table 3.

*Response: We agree with Reviewer #1’s comment. Indeed, we have already acknowledged the limitations of coproantigen ELISA for the detection of G. duodenalis in current lines 566-568 of the Discussion section. Please also note that this assay was the screening method used for the detection of the parasite in the original GEMS project (see current lines 180-184 and associated references #36 and #42). Please also note that only samples with a positive result by coproantigen ELISA were used for genotyping and sub-genotyping purposes, as clearly stated in current lines 190 and 214.*

11. I am not going to refer to your results obtained when you include adults because that is not the purpose of the study. I include the limitations and discussion of your study. Of course, giardiasis is a parasite of public health importance. But trying to give an explanation for the presence of diarrhoea with the genotype without any other clinical data is not possible, it is also fully demonstrated that there is no relationship for the plasticity of the *Giardia* genome. Making conclusions about transversion and mutations is very risky, you have to be very careful about any change in SNPs that involve an amino acid and the change of a single nucleotide is not enough. Their results showed that in 8 sequences there was no change. It is difficult to follow it because it finally concludes that group B did not present transversion or mutations within group B (TABLE S 4).

*Response: we are unsure about what Reviewer #1 means in his/her comment. Regarding the association between a given genotype of the parasite and the occurrence of diarrhoea, please note that this issue has been one of the hottest topics of debate in the field of giardiosis in recent years, as clearly stated in the 7^th^ International Giardia and Cryptosporidium Conference hosted in Rouen (France) in 2019 (see Widmer et al. Parasite. 2020;27:14. doi: 10.1051/parasite/2020011). Historically, the main problems of the studies conducted to investigate this issue were the relatively low number of samples genotyped and the improper recruiting of participants. Please note that both issues have been sorted out in the present study, where a large (n = 222) panel of individuals of paediatric age (recruited as cases and controls paired by sex and age) were available and fully typed. As commented in several parts of the manuscript (e.g. lines 485-489 and 566-568 of the Discussion section) this is exactly the main strength of the present study.*

*Regarding the identification of transition and transversion mutations involving amino acid change, please note that this was determined by translating the nucleotide sequence into an amino acidic sequence using specific software. We were able to identify single mutations that clearly involved changes in amino acid at the protein level. In the case of ambiguous positions (double peaks) please note that we considered the two traces detected at each ambiguous site, only reporting the potential amino acid change in those cases where it was possible. All these data were clearly stated in a specific column (labelled as “Amino acid change”) in S3-S5 Tables. Please also note that most of the mutations detected corresponded to transitions that involved no amino acid change (silent mutations). At present we do not know if the detected mutations involving amino acid change have physiological relevance, but this point is clearly out of the scope of the present survey.*

12. They conclude that the study represents an investigation trying to evaluate the genetic diversity of *Giardia* in children with and without diarrhea first point the genotype of the parasite was not correlated with diarrhea in children Which is demonstrated by finding in 9 out of 10 children agrees that it is not statistically significant The third point, the explanation of the diversity found in the *Giardia* III / *Giardia* IV genotypes must present the cleanest technique in its results without ambiguities, no double peaks and the purity of the DNA used.

*Response: Please also note that, in the case of the present study, the occurrence of ambiguous (double peak) positions are related to the high infection and re-infection rates of G. duodenalis (or to closely-related genetic recombination events) characteristic of a highly endemic area such as Mozambique rather than to the DNA quality and purity. This point has been already mentioned and discussed in lines 528-532 of the Discussion section. Please note that generated chromatograms at the three loci investigated were of good quality in most instances. This is exactly what allowed us to define the genetic variability of G. duodenalis with the precision shown in S3-S5 Tables. This point has been now highlighted in current line 319-320 of the Results section.*

13. The rationale for classifying *Giardia* into genotypes should be specified, because changes in electropherograms with a single nucleotide are not enough. The presence of ambiguities and double peaks that do not give solidity to these results, generally this occurs when lack of purity in the DNA to demonstrate the purity of the DNA.

*Response: Reviewer #1 raised an important issue. Please note that in the present study we defined genotype as a genetic variant within sub-assemblage level, as previously proposed by others (see Ryan and Cacciò Int J Parasitol. 2013;43:943–956). Observed genotypes are thoroughly described by alignment with well-known reference sequences, as clearly stated in current S3-S5 Tables. This very same terminology has been routinely used in previous human molecular epidemiological studies on G. duodenalis conducted by our research team at different geographical locations including Angola (Dacal et al. Parasit Vectors. 2018;11:67), Brazil (Seguí et al. Parasit Vectors. 2018;11:490), Ethiopia (de Lucio et al., PLoS One. 2016;11:e0159992), Iran (Kasaei et al., Parasitol Res. 2018;117:1425-1431), Spain (de Lucio et al. PLoS One. 2015;10:e0143981), and Turkey (Sarzhanov et al., Parasitology. 2020 Sep 28:1-45.), among others.*

*Regarding the question about the ambiguity of BIII/BIV sequences and the quality of the starting DNA material, please see our answer to comment #12.*

**Reviewer´s #2 comments**

This is a very important paper for the *Giardia* field, that in addition to bring insights on the lack of relationship between assemblage and clinical disease, performs a large-scale genotyping in samples from Mozambique, constituting an important source of epidemiological information for that country. After careful revision of the manuscript PNTD-D-20-01460 I have only some minor comments that might be useful to improve the current version.

*Response: We thank Reviewer #2 for her preliminary positive appraisal of our study.*

1. Table 2 demonstrates how the TAC qPCR was superior in sensitivity than the PCR performed in Mozambique. Could the authors please add in the Discussion whether the publication of this articles can impact – or not – changes in the national policy – if any-, for epidemiological characterization/reporting of *Giardia* infections.

*Response: We would like to clarify that at present routine monitorization of G. duodenalis at national scale is not conducted in Mozambique. This is mainly due to the lack of an effective way of detecting and notifying the incidence of the disease. In our opinion, the priority should be the design, implementation, and long-term maintenance of an effective national surveillance system able to monitor the presentation of new cases through time and with a good national coverage. Genotyping would be second in the prioritized task list.*

2. Do the authors have an explanation to why mixed infections were determined only by TAC qPCR but not with genotyping? If this is important, please add this response to the Discussion.

*Response: No, at present we do not have a clear explanation for this finding. It is possible that differences in primer design and diagnostic sensibilities of the PCR methods may be involved. We have now acknowledged these possibilities in lines 566-568 of the Discussion section.*

3. I would remove lines 506 to 512 - we do not want to speculate findings for other assemblages if the assay was designed to focus more on detection of assemblages A and B.

*Response: We disagree with Reviewer #2 in this matter. Please note that the initial assignment of assemblages in this survey was based on the results obtained by conventional multiplex PCR. This method was specifically developed to detect (and differentiate among) assemblages A and B, the two G. duodenalis genetic variants predominant in humans. MLSG studies were also based on samples that tested positive by conventional multiplex PCR. In practical terms this means that no specific method was used to investigate the presence of G. duodenalis assemblages other than A and B, so we cannot guarantee that assemblages C-F would be detected if present in the children population surveyed. We believe that this important point should be highlighted in the Discussion section, as this study has predominantly assessed anthropically (but not zoonotically) transmitted G. duodenalis assemblages. Therefore, no further changes have been introduced in the text.*

4. Abstract: at line 28, please clarify to the wide readership of PNTD what are “cases” in the GEMS study.

*Response: We clarified by adding in the sentence “…among controls than in diarrhoeal cases (>= 3 loosing stools in previous 24 hours)”.*

5. There is a typo at line 67

*Response: We carefully revised line 67 of the manuscript and could not find any. We will appreciate if the reviewer can indicate the exact word where the typo was detected to allow appropriate correction.*

6. Lines 109, 110: the following sentence is not necessary in my opinion: “which are referred by using Roman numerals as suffixes”.

*Response: The sentence has been removed as suggested by Reviewer #2.*

7. Lines 163 to 167 -Please clarify or give more detail to the reader: if the author said that there were 3 years of GEMS1 + 1 year of GEMS1A, please explain how this study consisted in 5 years.

*Response: We acknowledge the comment from reviewer 2 and have clarified how the 5 years of data were achieved in lines 165 – 169. Indeed, for all other sites, GEMS consisted of 3 years for GEMS1 + 1 year for GEMS1A, however, for the Manhiça site (where the samples in this study were collected), an additional year, after GEMS1 and before GEMS1A, was included to assess the role of HIV on diarrhoeal aetiology and outcome (see Acácio et al.,* 2018 *doi: 10.1016/j.ijid.2018.05.012), therefore GEMS1 was conducted from December 2007 to October 2011, and GEMS1A from November 2011 to November 2012*

8. Line 77, please remove “as”.

*Response: Please note that the word “as” is not present in current line 77. We have used this term in the expression “…acting as suitable reservoirs…” in line 79, but in this case the term is correct.*

9. Please replace, after the first appearance, *Giardia duodenalis* for *G. duodenalis*, to make the reading sound smoother. Also, it will standardize the text.

*Response: After first appearance of the term Giardia duodenalis, the term G. duodenalis has been indicated in the text except when present at the beginning of a new sentence or in the title of a Table or Figure. In the latter cases we used the full name Giardia duodenalis.*

10. In Figure 1, “positive” is misspelled in one of the boxes

*Response: Corrected as per requested by Reviewer #2.*

11. Line 195, replace “was” for “were”

*Response: Corrected as per requested by Reviewer #2.*

12. Line 315 – please define the abbreviation here in the title

*Response: MLSG stands for multi-locus sequence genotyping. Corrected as per requested by Reviewer #2.*

13. Sentence at line 362 “Hotspots were…” can be rephrased for clarity.

*Response: Following reviewer #2 advice, the sentence has been rewritten as “Sites were SNPs tended to accumulate (defined by convenience as hotspots) included positions…”.*

14. Please choose “CHARACTERIZED” or CHARACTERISED”

*Response: the British English grammar has been used through the whole manuscript.*

15. Line 427 – Typo in “giardian”

*Response: Corrected as per requested by Reviewer #2.*

16. Line 500, “The above mentioned result is in line with those previously reported in most African countries” – I would remove this sentence or explain better what the authors are trying to say here.

*Response: Following Reviewer #2 advice, the sentence has been rewritten as “The above-mentioned result is in line with those previously reported in most African countries demonstrating the predominance of assemblage B over assemblage A”.*

17. Line 553 – missing “d” in sociodemographic

*Response: Corrected as per requested by Reviewer #2.*
